# What Can the Bacterial Community of *Atta sexdens* (Linnaeus, 1758) Tell Us about the Habitats in Which This Ant Species Evolves?

**DOI:** 10.3390/insects11060332

**Published:** 2020-05-28

**Authors:** Manuela de Oliveira Ramalho, Cintia Martins, Maria Santina Castro Morini, Odair Correa Bueno

**Affiliations:** 1 Centro de Estudos de Insetos Sociais—CEIS, Instituto de Biociências, Universidade Estadual Paulista, UNESP, Campus Rio Claro, Avenida 24A, 1515, Bela Vista, Rio Claro 13506-900, SP, Brazil; odaircb@rc.unesp.br; 2 Department of Entomology, Cornell University, 129 Garden Ave, Ithaca, NY 14850, USA; 3 Campus Ministro Reis Velloso, Universidade Federal do Piauí, Av. São Sebastião, 2819, Parnaíba, Piauí 64202-020, Brazil; martins.c@ufpi.edu.br; 4 Núcleo de Ciências Ambientais, Universidade de Mogi das Cruzes, Av. Dr. Cândido Xavier de Almeida e Souza, 200, Centro Cívico, Mogi das Cruzes 08780-911, SP, Brazil; mscmorini@gmail.com

**Keywords:** environment, extensive cultivation, bacterial diversity, sustainable development, conservation

## Abstract

Studies of bacterial communities can reveal the evolutionary significance of symbiotic interactions between hosts and their associated bacteria, as well as identify environmental factors that may influence host biology. *Atta sexdens* is an ant species native to Brazil that can act as an agricultural pest due to its intense behavior of cutting plants. Despite being extensively studied, certain aspects of the general biology of this species remain unclear, such as the evolutionary implications of the symbiotic relationships it forms with bacteria. Using high-throughput amplicon sequencing of 16S rRNA genes, we compared for the first time the bacterial community of *A. sexdens* (whole ant workers) populations according to the habitat (natural versus agricultural) and geographical location. Our results revealed that the bacterial community associated with *A. sexdens* is mainly influenced by the geographical location, and secondarily by the differences in habitat. Also, the bacterial community associated with citrus differed significantly from the other communities due to the presence of *Tsukamurella*. In conclusion, our study suggests that environmental shifts may influence the bacterial diversity found in *A. sexdens*.

## 1. Introduction

Several studies have focused on understanding the factors that influence bacterial communities and their implications for the host, ranging from mammals [1] to ants [2,3,4,5,6,7,8]. Previous studies on vertebrate and invertebrate animals, including insects, have shown that bacterial communities are influenced by different factors such as host phylogeny [5,9,10], stages of development [4,11] and host environment [3]. In addition, the diet [2,12] and age [13] are other important host attributes that should be considered in studies on associated bacterial communities. However, in order to fully understand the ecological patterns and mechanisms behind these naturally established symbioses, more studies exploring new hosts are needed.

The composition and ecological drivers of host bacterial communities have been extensively studied in ants of the genera *Camponotus* and *Cephalotes* [2,4,9,10,14,15]. These symbioses can be so intimate and fundamental to the participating organisms that in many cases, hosts cannot survive without their symbionts, in this case called the primary. Examples include *Blochmannia* associated with *Camponotus* species [3,4,16,17,18], and Rhizobiales with *Cephalotes* species [2]. However, because the Fomicidae family is very diverse on Earth and includes a large number of species with very different lifestyles [19], it is important to extend the study of the associated microbiota to other species [20].

Exclusively distributed in the Neotropical and Neartic regions, the genus *Atta* has 17 valid species and is most diverse in Brazil, where these ants are popularly known as leaf-cutting ants, with nine species recorded [19,21]. The evolutionary success of the genus is largely attributed to the mutualistic relationship with the fungus *Leucogaricus gongylophorus* and other associated organisms such as bacteria [22,23,24,25,26,27]. Leaf-cutter ant workers cut leaves and other pieces of plant material and use them as a substrate for the fungus symbiont, causing great economic damage mainly in extensive agricultural habitats [26,28]. There are estimates that the economic damage caused by leaf-cutting ants reach a scale of billions of dollars in the global economy [29]. Most control techniques have temporary effects and, in some cases, otherwise negatively impact the environment, as is the case for the chemical method that is most frequently utilized by farmers [30,31]. Among the leaf-cutting ant species, *Atta sexdens* (Linnaeus, 1758) (Formicidae: Myrmicinae) stands out in terms of abundance and wide distribution in Brazil [32] and the intimate association of leaf-cutting ants with human agriculture establishes this genus as an important group of ants to be studied. However, there are only a few studies about the ecological factors that can affect host bacterial communities in any ant genus [1,3,4,10,33,34], and even fewer that address these questions in *Atta* [23,35,36].

São Paulo state (Brazil) hosts a great diversity of natural and agricultural habitats in close proximity to each other, which makes it the ideal place to study the effect of these different habitats on the bacterial community of *Atta sexdens*. The Cerrado (Brazilian Savannah) and Atlantic Forest are the dominant natural habitats in the São Paulo state, but they are also heavily affected by agricultural practices. The Cerrado is characterized by trees with contorted trunks ranging from evergreen to deciduous in the dry season and large shrubs of about 2–8 meters in height, and a grassy ground [37]. The remaining natural coverage of the Cerrado in the São Paulo state is about 13% of its original (which corresponded to 33% of the state), highlighting the intense anthropogenic impact on its coverage in the state [38]. The Atlantic Forest is characterized by being an Ombrophilous Dense Forest [39] with several endemic species of flora and fauna. Historically, this habitat covered an extensive part of the Brazilian coast, and today it is reduced to just 12% of the original size [40]. In São Paulo state, the Atlantic Forest is reduced and now its remnants cover around 16.3% of the state [41]. The main agricultural habitats in the São Paulo state are sugar cane, followed in smaller scale by citrus and eucalyptus reforestation [42]. These activities use large quantities of pesticides, and fipronil and sulfluramide are specifically used for the control of leaf-cutting ants [28].

With the shift from natural to agricultural habitat, simplification of the landscape can occur due to floral simplification and application of insecticides. These changes can be beneficial to some insects and harmful to others [43]. In the case of insect pollinators, it was shown that the loss of environmental diversity caused by the agricultural process impacts on the fitness and success of the insects’ survival [44]. This fitness loss in agricultural habitats can be attributed to changes in the microbiota that can compromise the health of the insect, as has been proven in some studies of bees [45,46]. Therefore, associated bacterial communities can be modified with environmental shifts [47], and anthropogenic modifications can have an impact on the composition of the microbiota [48]. However, a study has never been conducted regarding the impact of changes in habitat directly affecting the microbiota associated with *Atta sexdens*.

We hypothesize that differences in habitat (natural and agricultural) can influence the diversity of the bacterial community associated with leaf-cutting ants. Furthermore, analyzing the factors that alter the bacterial community can provide insights into understanding the general biology of the host. Thus, in this study we tested whether 1) natural (Cerrado and Atlantic Forest) and agricultural (sugar cane, citrus and eucalyptus) habitats, and 2) geographical location (West, Center, East, South and North, from São Paulo state in Brazil) can influence the *A. sexdens* microbiota. We expect all these factors to influence and explain the diversity of bacterial communities associated with this leaf-cutting ant.

## 2. Materials and Methods

### 2.1. Sample Collection

**Figure 1:**
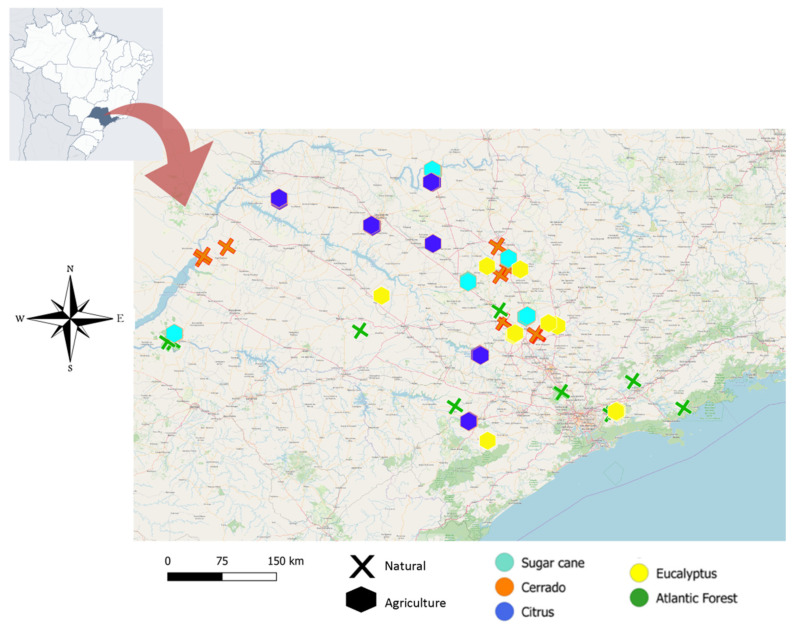
Samples of *Atta sexdens* collected from different natural and agricultural habitats.

Samples were collected in natural habitats (n = 4 areas of Cerrado, n = 5 areas of Atlantic Forest) and agricultural habitats (n = 5 sugar cane; n = 5 citrus; n = 6 eucalyptus), located in São Paulo state – Brazil (Figure S1). In each habitat, a linear transect was traversed along which foraging workers (with average size and weight between 10–20 mg and head width between 2.0 and 2.8 mm) were collected. The distance between colonies (n = 3 for each location) was at least 200 meters. We collected 12 workers in the Cerrado and 15 in the Atlantic Forest, 15 each in the sugar cane and citrus agricultural habitats, and 18 in the eucalyptus agricultural habitats (Figure 1; more details in Table S1). It is important to highlight that all agricultural habitats from this study are managed with agrochemicals: insecticides fungicides and herbicides for weed control. Synthetic fertilizers were also applied as part of cultural practices [42].

The taxonomic identifications were determined following morphological characters for species identification available in Mackay and Mackay (1986) [49] and vouchers were deposited in the collection de Mirmecologia do Alto Tietê. Samples used were collected in 95% ethanol in the field and kept at −20 °C until DNA extraction.

### 2.2. DNA Extraction and Bacterial DNA Sequencing

In total, we used 75 whole ant workers for DNA extraction using the DNeasy Power Soil Kit (Qiagen, Germantown, MD, USA) following recommended methods from Moreau [50] to avoid contamination. Furthermore, three negative controls were added to the extraction methods and to subsequent 16S rRNA amplifications and library sequencing. These negative controls are blank samples (corresponding to water instead of DNA in the polymerase chain reaction (PCR)) that were used to remove contaminants that may have been added in the process of obtaining the data. Amplification of the V4 region of 16S rRNA (515F/806R primers) was performed in triplicate following Earth Microbiome Project (EMP) protocols (http://www.earthmicrobiome.org/emp-standard-protocols/), also described in Caporaso [51]. PCR reactions followed recommendations from the Taq Platinum Kit (Invitrogen, Schwerte, Germany) with 2.5 μL Buffer 10×, 1.0 μL MgCl_2_ 50 mM, 1.0 μL dNTPs 10 Mm, 1.0 μL BSA (bovine serum albumin) 1 mg/mL, 0.5 μL 515F primer 10 uM, 0.5 μL 806R primer 10 uM, 0.5 μL Taq Platinum 5U/μL, 5 μL template DNA (10 ng/µL), 13.0 µL PCR Water (Certified DNA-free). The reaction was submitted to the thermal cycler under the following conditions: 95 °C for 3 min, 30 cycles at 95 °C for 30 s, 60 °C for 30 s and 72 °C for 30 s, with final of 72 °C for 10 min and 4 °C hold. Purification was performed with the Ampure X beads and adapter connections (NEXTERA XT) were also performed following the manufacturer’s recommendations. The samples were quantified via Nanodrop (Thermo Fisher Scientific, Waltham, MA, USA). Pooling and normalization were performed so that the samples had the same concentration. Then, the DNA pool was diluted down to 2 nM, denatured, and diluted to a final concentration of 16 pM with a 20% PhiX for sequencing on the Illumina MiSeq with run 2 × 250 pb at the Centro de Genômica (ESALQ, Piracicaba, SP, Brazil). All raw sequence data are publicly available in NCBI SRA accession number PRJNA556881 and study SAMN12373152.

### 2.3. Bioinformatic Analysis

Data were imported into Qiime2 [52] following the Casava 1.8 paired-end demultiplexed fastq protocol. The Dada2 plugin was used to create a feature table and quality control [53,54]. Paired-end sequence reads were trimmed (forward reads trim 30 and trunc 245, reverse reads trim 30 and trunc 220) for better results and SILVA_132_QIIME database [55,56] with 99% similarity was used to access ASVs (amplicon sequence variants) for the taxonomic information from the data sequenced. We trained our specific classifier based on our sample preparation and sequencing, using the “feature-classifier classify-sklearn” command [57].

Negative controls were filtered by the prevalence method using the Decontam package in R software (R Development Core Team, Vienna, Austria, 2019) following default settings [58]. Once our feature table was filtered, an extra filtering step was applied in Qiime2 in order to remove mitochondrial, chloroplast, and low abundance ASVs. Reads were aligned with the “align-to-tree-mafft-fasttree” command [59] and the phylogeny was inferred before alpha and beta diversity analyses [60,61]. Samples were normalized at 2000 reads in subsequent analyses.

The categories used in order to test for potential influences in leaf-cutting ants were conducted in two classes: 1) natural habitats (Cerrado and Atlantic Forest) and main agricultural habitats (sugar cane, citrus and eucalyptus), and 2) geographical location where the colonies were collected. To test the influence of geographical location within São Paulo State (Brazil), we divided the data according to their cardinal points of collection: West, Center, East, South, and North of the state and samples were assigned to those locations independent of their status belonging to natural or agricultural habitats (Figure S1). Permutational multivariate analysis of variance (permanova) tests with the “*p*-pairwise” parameter were conducted with Unifrac distance, a qualitative measure that includes phylogenetic relationships between features [62]. Analysis of non-metric multidimensional scaling (NMDS), principle coordinates analysis (PCoA), and related statistics were implemented to better visualize our results of relationships between ecological communities and in the PAST3 software package [63,64,65] and Qiime2 in Emperor software [61]. Similarity percentage (SIMPER) analysis [63] was used to verify the ASVs’ contribution to structuring the bacterial community found in the study. Network Analysis and heatmap plots of bacterial communities were conducted in R software [66] with the packages phyloseq [67] and ggplot2 [68].

### 2.4. Data Accessibility

All raw sequence data are publicly available in NCBI SRA accession number PRJNA556881 and study SAMN12373152.

## 3. Results

Samples were normalized to a minimum of 2000 reads per sample with sequencing reaching the expected depth (Figure S2). After this cutoff, 75 samples remained and represented a total of 6,383,699 reads distributed in 273 different ASVs. Sampling ranged from a maximum of 177,158 reads to a minimum of 2244 reads, confirming the successful sequencing of the bacterial community associated with *A. sexdens*.

**Figure 2:**
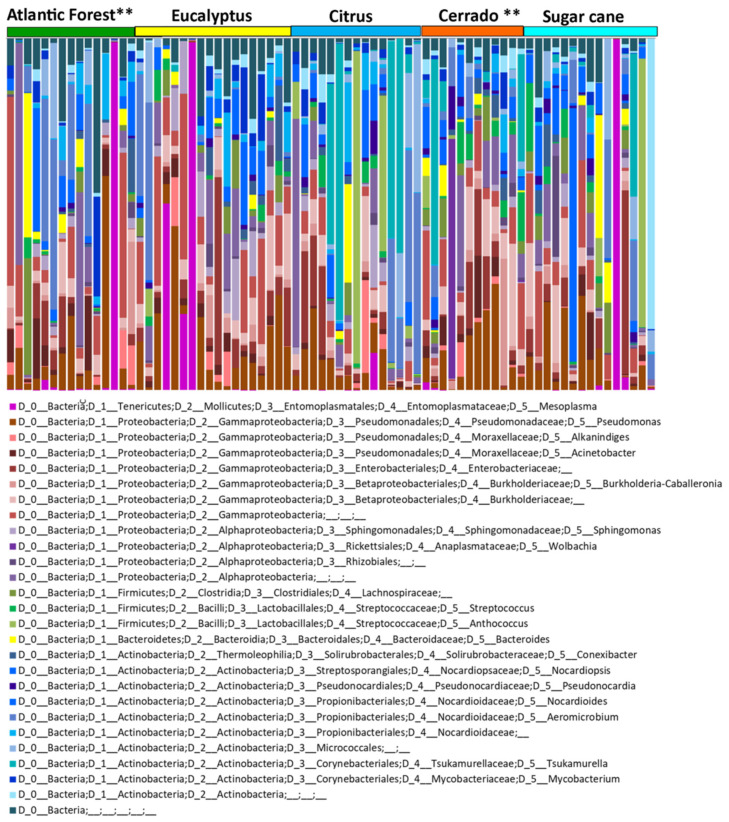
Summary of main microbiome amplicon sequence variants (ASVs) found in *Atta sexdens* with 16S rRNA amplicon sequencing. Alpha diversity of microbiota in each sample of *A. sexdens*. Samples were grouped according to the habitat (natural or agricultural) to which they belong. Bar graphs for each library (one column = community from a sample) show the abundance of sequence reads classified to selected 99% similarity. Each color represents a distinct bacterium. ** indicates samples in natural habitat.

Subsequent analyses were performed at the family taxonomic level of the bacterial communities or at the high hierarchy taxonomic level when available in the database. The ASV table and the overview of relative abundance can be seen in Table S2 and Figure S3, respectively. The main bacterial community recovered revealed *Mesoplasma* as the predominant bacterial genus (7%), followed by *Tsukamurella* (6%), *Aeromicrobium* (Nocardioidaceae) (5%) and others in smaller quantities. Overall when we include all natural and agricultural habitats, the bacterial community of *A. sexdens* consists of several taxa per sample (Figure 2), which makes it difficult to detect any bacterial structure or community patterns.

### 3.1. Beta Diversity and the Influence of Natural and Agricultural Habitats

**Figure 3:**
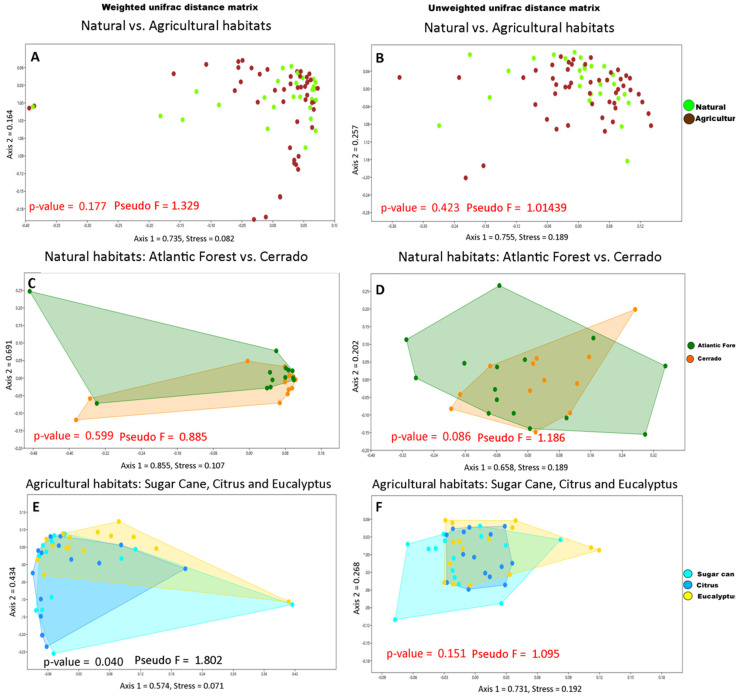
Non-metric multidimensional scaling (NMDS) plot illustrating bacterial community structure among *Atta sexdens* workers of natural vs. agricultural habitats. (**A**). Weighted unifrac distance matrix for samples from natural and agricultural habitats. (**B**). Unweighted unifrac distance matrix for samples from natural and agricultural habitats. (**C**)**.** Weighted unifrac distance matrix for samples from Atlantic forest and Cerrado. (**D**)**.** Unweighted unifrac distance matrix for samples from Atlantic Forest and Cerrado. (**E**)**.** Weighted unifrac distance matrix for samples from agricultural habitats. **(F**)**.** Unweighted unifrac distance matrix for samples from agricultural habitats. Dots were colored according to the different groups. The distance between bacterial communities represents their underlying distance in the multivariate space. Statistics highlighted in red show p values greater than 0.05. Besides one significant p value in the weighted unifrac distance for the agricultural habitat, note that there is not a clear structuring of the different groupings.

The results of the beta diversity analysis suggest that the bacterial community of leaf-cutting ants do not differ between natural habitats and agricultural habitats (permanova weighted unifrac distance: *p*-value = 0.177, pseudo F = 1.329, Figure 3A; permanova unweighted unifrac distance: *p*-value = 0.423, pseudo F = 1.014, Figure 3B). When beta diversity analysis was conducted only with samples from natural habitats, we also did not find statistically significant differences (permanova weighted unifrac distance: *p*-value = 0.599, Pseudo F = 0.858, Figure 3C; permanova unweighted unifrac distance *p*-value = 0.086, Pseudo F = 1.186, Figure 3D). Overall, there is no difference in the bacterial community of *A. sexdens* associated with the Cerrado or Atlantic Forest.

When analyzing only leaf-cutting ant bacteria associated with agricultural habitats (cane, citrus and eucalyptus) the beta diversity results indicated an influence in the weighted bacterial community (permanova weighted unifrac distance: *p*-value = 0.04, Pseudo F = 1,802) (Figure 3E). Unweighted analysis differences were not significant (permanova unweighted unifrac distance: *p*-value = 0.151, Pseudo F = 1.095) (Figure 3F), confirming that the abundance of bacteria recovered in weighted unifrac distance are important in helping explain associations with agricultural habitats, but not the bacterial composition.

**Figure 4:**
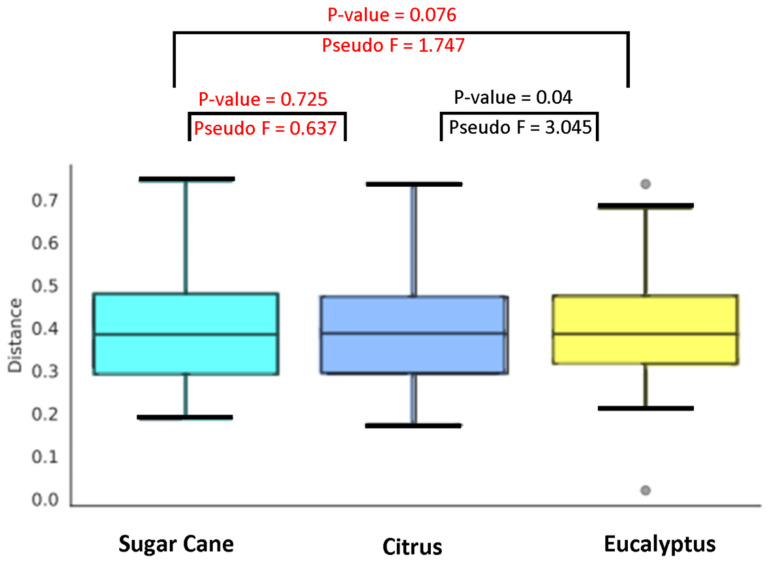
Pairwise permutational multivariate analysis of variance (permanova) results of agricultural sites. Results highlighted in red indicate p value greater than 0.05. Bacterial communities associated with citrus and eucalyptus agriculture contributes to the difference found in Figure 3.

**Figure 5:**
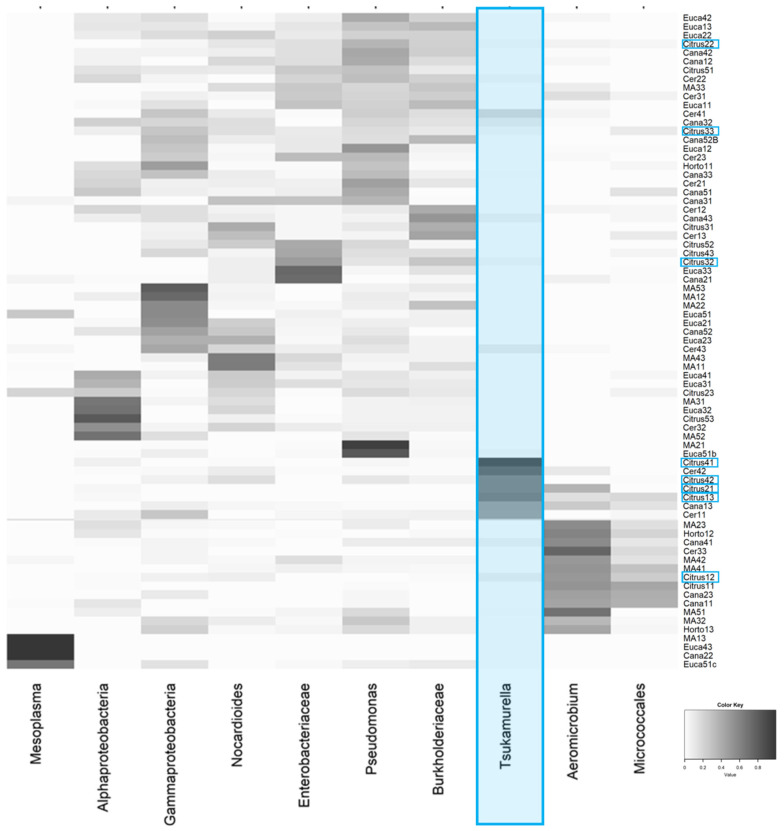
Main bacterial ASVs found in *Atta sexdens* are visualized in this heatmap. The shading in the heatmap indicate variation in the relative abundance of different bacteria in all samples from this study, ranging from 0% (white) to 100% (black). Highlighted in blue is the *Tsukamurella* bacteria, which although found in different habitats, is mainly associated with citrus (blue boxes).

To explore this result more deeply, we performed the paired permanova test (weighted unifrac distance) that allows all samples associated with the agricultural habitat to be analyzed in pairs (see Figure 4). The only pair that obtained a significant result was of bacterial communities associated with citrus and eucalyptus agricultural habitats (*p*-value = 0.04 and Pseudo F = 3.045), contributing to the difference found in Figure 3. In addition, through the results of SIMPER analysis, the main bacteria (ASVs) associated with citrus and eucalyptus agriculture are identified in Table 1 (for all groupings see Table S3). Note that *Tsukamurella* is responsible for 12.5% of the difference found in this pair (citrus and eucalyptus), followed by *Aeromicrobium* with 7.07% and others in smaller abundance. Our heatmap analysis gives a better view of *Tsukamurella*’s association with *A. sexdens*, especially citrus (Figure 5).

### 3.2. Beta Diversity and the Influence of Geographic Location

**Figure 6:**
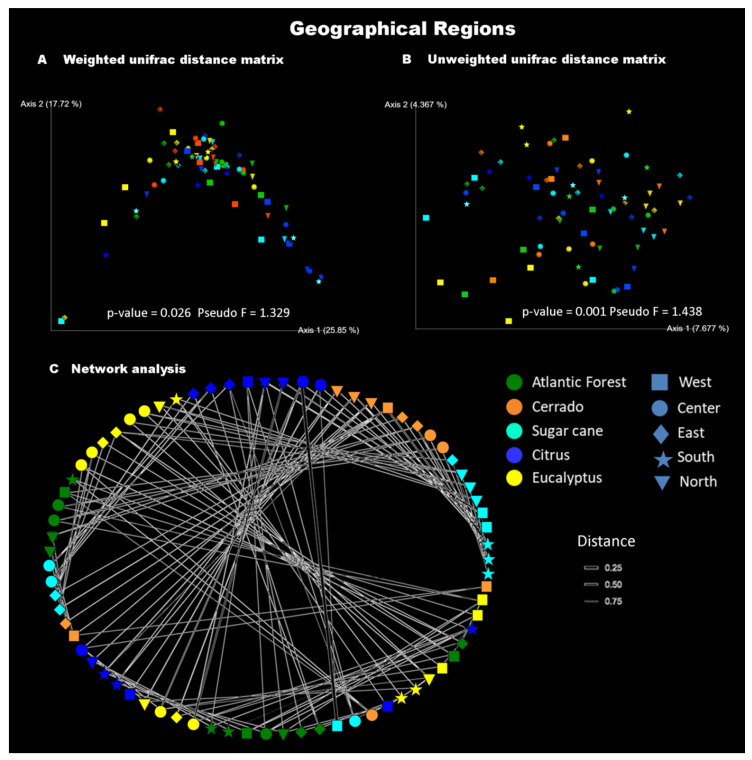
Plot illustrating bacterial community structure among *Atta sexdens* workers of different regions (West, Center, East, South, and North), and habitats (natural and agricultural). (**A**). Principal coordinates analysis (PCoA) with weighted unifrac distance matrix. (**B**). PCoA with unweighted unifrac distance matrix. (**C**). Network analyses of bacteria present in *Atta sexdens* workers in different habitats. Note the complexity of the bacterial communities associated with leaf-cutting ants. Colors represent different habitats, and shapes represent different geographical regions.

Another factor that may be responsible for influencing the diversity of bacterial communities is the locality where the host was collected. Our results show the influence of the geographic regions of the state of São Paulo (Brazil) (West, Center, East, South, and North) on the sampled bacterial communities in terms of abundance (permanova weighted unifrac distance: *p*-value = 0.026, Pseudo F = 1.534) and presence/absence (permanova unweighted unifrac distance: *p*-value = 0.001, Pseudo F = 1.438). That is, there is a difference not only in the composition but also in the abundance of bacterial communities associated with leaf-cutting ants when considering the geographic regions of the state of São Paulo (Brazil) (Figure 6A,B). The network analysis shows the complexity of the bacterial community associated with *A. sexdens* (Figure 6C).

We also tested for any significant differences between the habitats (natural: Cerrado and Atlantic Forest; and agricultural: sugar cane, citrus and eucalyptus) present within each geographic region. For the Center region we found significant results in both the abundance analysis and composition analysis (which tests for presence or absence) (Table 2). For the other regions analyzed separately, our results show that there is a significant difference in bacterial composition (presence and absence) associated with different habitats, but not in abundance.

When we conducted analyses between natural and agricultural habitats, although we were able to reveal interesting results regarding the *Tsukamurella* influence mainly associated with citrus, some data were scrambled and diluted in the vast amount of information generated by this technique. When first investigating the influence of geographic location (West, Center, East, South and North), we determined that it does have a large impact on the weighted and unweighted composition of the bacterial community associated with *A. sexdens.* However, when the analyses were conducted by region (separately), the results showed significant differences in the composition (unweighted) of bacterial communities associated with different habitats, i.e., natural (Cerrado and Atlantic Forest) and agricultural (sugar cane, citrus and eucalyptus).

**Figure 7:**
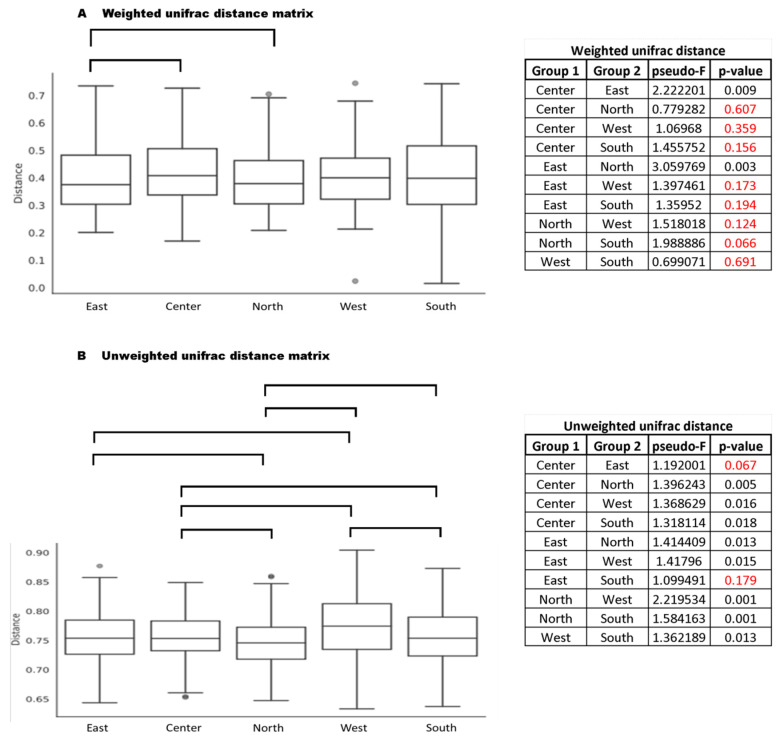
Pairwise permanova results of different regions. (**A**). Weighted unifrac distance matrix. (**B**). Weighted unifrac distance matrix. Results highlighted in red indicate *p* values greater than 0.05. Bars represent the pairs of regions with significant differences.

To test which pairs of geographic regions contributed to the difference found in Figure 6, paired permanova tests verified abundance (weighted unifrac distance) and composition (unweighted unifrac distance) and found that several groupings showed significant differences (see Figure 7). In general, all regions (West, Center, East, South, and North) influenced the abundance and composition of bacterial communities associated with this leaf-cutting ant. Therefore, the pairs that obtained significant results were subjected to SIMPER analysis, where we obtained the main bacteria associated with these groupings (peer regions) (see Table 3). Our results are consistent, showing that *Tsukamurella* is one of the major bacteria responsible for differences in all groupings of geographic regions. These results were also confirmed by the analysis in Table 1, further supporting that this bacterium has a significant impact on the bacterial community of *A. sexdens*. In addition, *Mesoplasma*, the main bacterium found in the present study (see Figure 2), was also frequent and one of the major bacterial genera responsible for the differences found in almost all clusters, except Center vs. North.

### 3.3. Alpha Diversity: Different Habitats and Geographical Locations

Alpha diversity also presented distinct results when the analysis was conducted comparing only the different habitats and geographic regions with the Faith phylogeny index. According to a Kruskall–Wallis test, only geographic region influences the alpha diversity associated with *A. sexdens* (H = 16,795, *p*-value = 0.002). As this richness index is qualitative, it takes into account the composition of the bacterial community (presence and absence). However, the same analysis conducted with the Shannon index regardless of abundance did not yield any significant results (see Figure S4). Therefore, the different geographical regions may influence the composition of alpha diversity associated with *A. sexdens*, but do not appear to influence the abundance.

## 4. Discussion

There are several factors that can predict and influence the diversity (composition and abundance) of microbial communities associated with a given host [8]. The microbiota of leaf-cutting ants has already been addressed in several studies [23,27,34,35,36,69,70,71], but there remains much to understand about the drivers of these symbioses. This lack of knowledge motivates the present study, which is the first to compare the bacterial communities of ants in natural (Cerrado and Atlantic Forest) to agricultural (sugar cane, citrus and eucalyptus) habitats, as well as consider geographical location (West, Center, East, South, and North of the state of São Paulo-Brazil), to assess the factors that may influence microbial diversity. We found differences in microbiota of the different samples from the same species in different habitats and geographic locations. While each strain of bacteria can be acquired in a different way [72], our results may reflect the fact that these bacteria are being acquired from different environmental conditions (e.g., landscape simplification, different plant availabilities, agrochemicals, and soil), through horizontal more than vertical transmission. That is, even with sophisticated defense systems and behavior utilized by *A. sexdens* [49,73,74], the bacterial communities of the present study were altered mainly due to differing geographical locations, and secondarily due to natural and agricultural habitats.

There are several studies that show that the composition and abundance of the microbiota is impacted by horizontal acquisition [3,47,70,75,76,77]. Our study corroborates those that have found that the geographic location of the host influences the diversity of the bacterial community, whether in the case of mammals [75], birds [78,79], other insects [47,77,80], or also of ants [3,70,76,81]. For example, the bacterial diversity recovered from the mesosoma and head of *Camponotus* workers is influenced by the habitat where these ants were collected, which suggests that these bacteria are being acquired via horizontal transfer [3]. Another study that reinforces the impact of geographical location on the diversity of bacterial communities was conducted by Martins and Moreau [76] with *Pheidole* species collected around the world. The same pattern of acquisition of microbial communities was also reported in fourth-instar larvae of *Solenopsis invicta* ants [81] and the lower attine species *Mycocepurus smithii* [70].

*Atta cephalotes* workers also showed a diverse and unstable microbiota with Mollicutes being the main bacteria recovered [36]. The bacterial community recovered from *A. sexdens* is also very complex, and this study was able to identify several important bacterial taxa associated with this species. ASVs identified as *Mesoplasma* sp. (Mollicutes, Entomoplasmatales) is one of the most prevalent bacteria in our sample with 7% relative frequency. Our results corroborate a previous study [23] that reports evidence of this bacterium mainly associated with the post-pharyngeal gland (70%) and also in smaller quantities in the midgut of the queen and the whole body of the worker of *A. sexdens*. Meirelles et al. [35] describes the association of *Mesoplasma* sp. with infrabucal pellets carried by *A. texana* queens. Also in this study, through phylogenetic analysis, the authors show specificity of this bacterium associated with eight different genera of Attine, since it formed a monophyletic clade. *Mesoplasma* sp. associated to the army ants [82] was also grouped in another clade, also showing specificity to army ants [83]. The function of this ant-associated bacterium was investigated with the genome of two strains, and it was found that each strain was acquired independently from a different origin. These bacteria are capable of decomposing arginine and providing nitrogen-rich amino-acid [84]. In that same study, the authors also concluded that the relative abundance of *Mesoplasma* sp. is related to the type of substrate that the ant worker offers to the fungus garden. Our study found a high relative abundance of these bacteria, which suggests that it must have been facilitated by the substrate of *A. sexdens*.

The next most abundant ASV recovered in the present study is *Tsukamurella* sp. (Actinobacteria), which despite occurring in other habitats was strongly related to the citrus agricultural habitat. Although its function is not fully understood, there are studies reporting that these bacteria may be an endophytic growth promoter [85] minimizing the growth of phytopathogenic fungi [86], and also may act as a nematicide [87] used in agriculture. The third most common ASV was identified as *Aeromicrobium* sp. (Actinobacteria), which has also been reported on species of ants as well [88] but is generally associated with the soil [89]. Other studies have reported the presence of ant-associated Actinobacteria, especially the genus *Pseudonocardia* sp., often associated with other Attines ants [71,90]. These bacteria are capable of producing compounds with antibiotic properties that defend the fungal garden and the ant colony from disease [25,71,90]. The samples of *A. sexdens* from our study were also associated with these bacteria in smaller quantities compared to the literature, which suggests that perhaps *A. sexdens* is also relying on other measures to maintain the health of the fungus garden and the colony. Further studies are needed to address this issue.

The phylum Proteobacteria has also been reported in *A. cephalotes* Linnaeus 1758 and *A. colombica* Guérin-Méneville 1844 [69,91]. *Pseudomonas* and Burkonderialles are bacteria belonging to this phylum, and were associated with the workers of leaf-cutting ants of the present study, although in smaller abundances. Some bacteria present in the insect microbiome may be able to metabolize agrochemicals, making the host pesticide-resistant [92,93,94], and it has been shown that certain strains of the genus *Pseudomonas* sp. are associated with insect resistance to insecticides [95]. The bacterial community is subject to several environmental stressors that may alter its composition [96]. As an example, certain strains of *Burkholderia* sp. are capable of degrading insecticide fenitrothion (organophosphate) [97]. Tago et al. [98] found an increase of this bacterium in the soil after the application of the pesticide in sugar cane agricultural habitats.

In another study of *Acromyrmex* spp., besides Entomoplasmatales, the bacteria *Wolbachia* and Rhizobiales were highlighted in association with these ants. These latter two bacteria are highly associated with several genera of ants [2,8,36,99]. While the function of *Wolbachia* associated with ants is still unknown, it is transmitted vertically, although its diversity is influenced by geography [17,36,100]. On the other hand, Rhizobiales is also often associated with nitrogen fixation and recycling [2,69], which is extremely important especially in the case of herbivorous ants. Our results also recovered both bacteria associated with *A. sexdens*.

The microbiota associated with a host can be composed of mutualists, commensals and pathogens, and many of the bacteria associated with *A. sexdens* are probably transient bacteria that have no associated role in ants. With the 16S rRNA amplicon technique, we cannot be sure of the microbe–host relationships or their functions. However, overall these bacteria may have been acquired horizontally and thus may reflect the environment (habitats). In addition, although the bacterial community of *A. sexdens* is unstable and highly variable, we do not know how this complexity varies in the different castes within the colony, and its implications for the host. In the case of *Atta cephalotes* it was found that the bacteria Mollicutes passes via social transmission of workers to the larvae [36]. Future studies that consider the different castes and location of this microbiota in the ant body may help to elucidate the mode of transmission and consequently the function of these bacteria associated with *A. sexdens*.

## 5. Conclusions

Genomic approaches have become increasingly accessible and can be used as excellent tools to explain several biological phenomena such as intra- and interspecific relationships, species ecology such as diet and symbioses, as well as molecular, physiological, behavioral and evolutionary features of the host. These approaches have the potential to generate data that can contribute to the understanding of symbiotic interactions within the field of myrmecology [2,101,102,103,104,105]. Recent next-generation sequencing techniques have made it possible to analyze the communities of bacteria present in several hosts. Specifically for ants, many studies have demonstrated the importance of these symbionts and how they can influence host attributes [2,6,8,10,20,106]. However, studies of microbial interactions are challenging because they involve dynamic systems that can be shaped by the sum of abiotic factors, as presented here. This is the first study that compared the bacterial communities of *A. sexdens* in natural habitats, such as Cerrado and Atlantic Forest, to those in agricultural habitats (sugar cane, citrus and eucalyptus) and that considered geographic location (West, Center, East, South, and North of the state of São Paulo-Brazil), showing that all these factors may influence microbial diversity. Knowledge about this insect-associated microbiota may help to develop strategies to increase insect pest resistance in agricultural habitats, as well identify bacteria that can act as bioremediation of pesticides and help recover degraded areas [107].

## Figures and Tables

**Table 1:** Similarity percentage (SIMPER) analysis of main bacteria in citrus and eucalyptus group. This test indicates the contribution of specific ASVs to the observed differences in community structure among citrus and eucalyptus. Note that for this group, *Tsukamurella* is the most influential bacteria.

Habitats	Overall Average Dissimilarity	Most Influential ASV/Taxonomy	Percent Contribuition to Difference	Cumulative %
*Citrus* vs. *Eucalyptus*	85.00%	Tsukamurella	12.05	12.05
		Aeromicrobium	7.07	19.12
		Anthococcus	5.89	25.01
		Mesoplasma	5.11	30.12
		Micrococcales	4.3	34.42
		Alphaproteobacteria	3.91	38.32
		Enterobacteriaceae	2.84	41.17
		Nocardioides	2.66	43.83
		Pseudomonas	2.53	46.36
		Gammaproteobacteria	2.09	48.45
		Burkholderiaceae	2.04	50.49

**Table 2:** Abundance (permanova weighted unifrac distance) and composition (permanova unweighted unifrac distance) analysis of the microbiota associated with *A. sexdens* collected at different geographical locations. This table shows significant differences mainly in the composition between the habitat (natural: Cerrado and Atlantic Forest; and agricultural: sugarcane, citrus and eucalyptus) in each geographical location.

Regions	Permanova Weighted Unifrac Distance		Permanova Unweighted Unifrac Distance	
	*p*-Value	Pseudo F	*p*-Value	Pseudo F
Center	0.025	1.940	0.001	1.162
East	0.254	1.127	0.018	1.212
North	0.494	0.977	0.001	1.192
West	0.075	1.43	0.014	1.280
South	0.376	1.092	0.016	1.198

**Table 3:** SIMPER analysis of main bacteria contributing to differences between each region pair. This test indicates the contribution of specific ASVs to the observed differences in community structure among different groups. Note that *Tsukamurella* remains the most influential bacteria, present in all clusters.

Regions	Overall Average Dissimilarity	Most Influential ASV/Taxonomy	Percent Contribution to Difference	Cumulative %
Center vs. East	74.77%	Alphaproteobacteria	10.63	10.63
		Aeromicrobium	8.38	19.01
		Gammaproteobacteria	7.64	26.65
		Mesoplasma	6.925	33.58
		Pseudomonas	6.403	39.98
		Tsukamurella	5.928	45.91
		Micrococcales	5.739	51.65
East vs. North	75.89%	Alphaproteobacteria	8.30	8.30
		Gammaproteobacteria	8.14	16.44
		Aeromicrobium	7.96	24.40
		Tsukamurella	7.43	31.83
		Mesoplasma	7.30	39.13
		Nocardioides	6.23	45.36
		Pseudomonas	4.92	50.28
Center vs. North	73.01%	Aeromicrobium	12.93	12.93
		Tsukamurella	11.34	24.27
		Micrococcales	6.81	31.08
		Gammaproteobacteria	6.52	37.60
		Nocardioides	5.188	42.79
		Alphaproteobacteria	4.634	47.42
		Pseudomonas	4.315	51.74
Center vs. West	82.17%	Tsukamurella	11.01	11.01
		Aeromicrobium	9.871	20.88
		Enterobacteriaceae	7.052	27.93
		Mesoplasma	6.649	34.58
		Micrococcales	6.587	41.17
		Alphaproteobacteria	6.284	47.45
		Bacteria	5.37	52.82
Center vs. South	79.77%	Aeromicrobium	8.51	8.51
		Mesoplasma	8.15	16.66
		Anthococcus	8.05	24.70
		Tsukamurella	7.87	32.57
		Micrococcales	5.74	38.31
		Burkholderiaceae	5.10	43.41
		Pseudomonas	4.73	48.14
		Alphaproteobacteria	4.57	52.71
East vs. West	82.48%	Mesoplasma	12.77	12.77
		Alphaproteobacteria	10.09	22.86
		Enterobacteriaceae	7.458	30.32
		Tsukamurella	6.881	37.20
		Gammaproteobacteria	6.798	44.00
		Pseudomonas	5.869	49.87
		Bacteria	4.881	54.75
North vs. West	81.97%	Tsukamurella	11.83	11.83
		Aeromicrobium	9.297	21.13
		Mesoplasma	6.928	28.06
		Nocardioides	5.837	33.89
		Gammaproteobacteria	5.473	39.37
		Enterobacteriaceae	5.438	44.8
		Bacteria	4.399	49.2
		Nocardiopsis	4.18	53.39
North vs. South	80.48%	Anthococcus	9.13	9.13
		Tsukamurella	8.84	17.98
		Mesoplasma	8.41	26.39
		Aeromicrobium	8.16	34.55
		Nocardioides	5.86	40.41
		Gammaproteobacteria	5.39	45.79
		Burkholderiaceae	4.52	50.31
West vs. South	86.00%	Mesoplasma	13.33	13.33
		Tsukamurella	8.571	21.90
		Anthococcus	8.282	30.18
		Enterobacteriaceae	5.651	35.83
		Aeromicrobium	5.253	41.08
		Burkholderiaceae	4.228	45.31
		Bacteria	4.195	49.51
		Pseudomonas	4.104	53.61

## Supplementary Materials

The following are available online at https://www.mdpi.com/2075-4450/11/6/332/s1, Figure S1: Location of *Atta sexdens* collection areas in São Paulo State, Brazil, Figure S2: Rarefaction curves were used to estimate richness in the Shannon index for 16S rRNA. The vertical axis shows the Shannon index observed and the number of sequences per sample is shown on the horizontal axis, Figure S3: The overview of bacteria relative abundance recovered from this study of *Atta sexdens*, Figure S4: Alpha diversity associated with *Atta sexdens*, Table S1: Samples of *Atta sexdens* collected from different natural and agriculture habitats, Table S2: Amplicon Sequence variants (ASVs) table highlighting each bacterium recovered from each sample of *Atta sexdens*, Table S3: SIMPER analysis of main bacteria in all differents habitats. This test indicates the contribution of specific ASVs to the observed differences in community structure among different groups. Note that *Tsukamurella* remains the most influential bacteria associated with citrus crop.
