# Supplementary material for: What Can the Bacterial Community of Atta sexdens (Linnaeus, 1758) Tell Us about the Habitats in Which This Ant Species Evolves?
Source: Insects. 2020 May 28;11(6):332. doi: 10.3390/insects11060332 (PMC7349130; doi:10.3390/insects11060332)
Supplement: Supplementary file 1 [file insects-11-00332-s001.zip › insects-751960-Supplmentary Materials/Figure S2.docx]

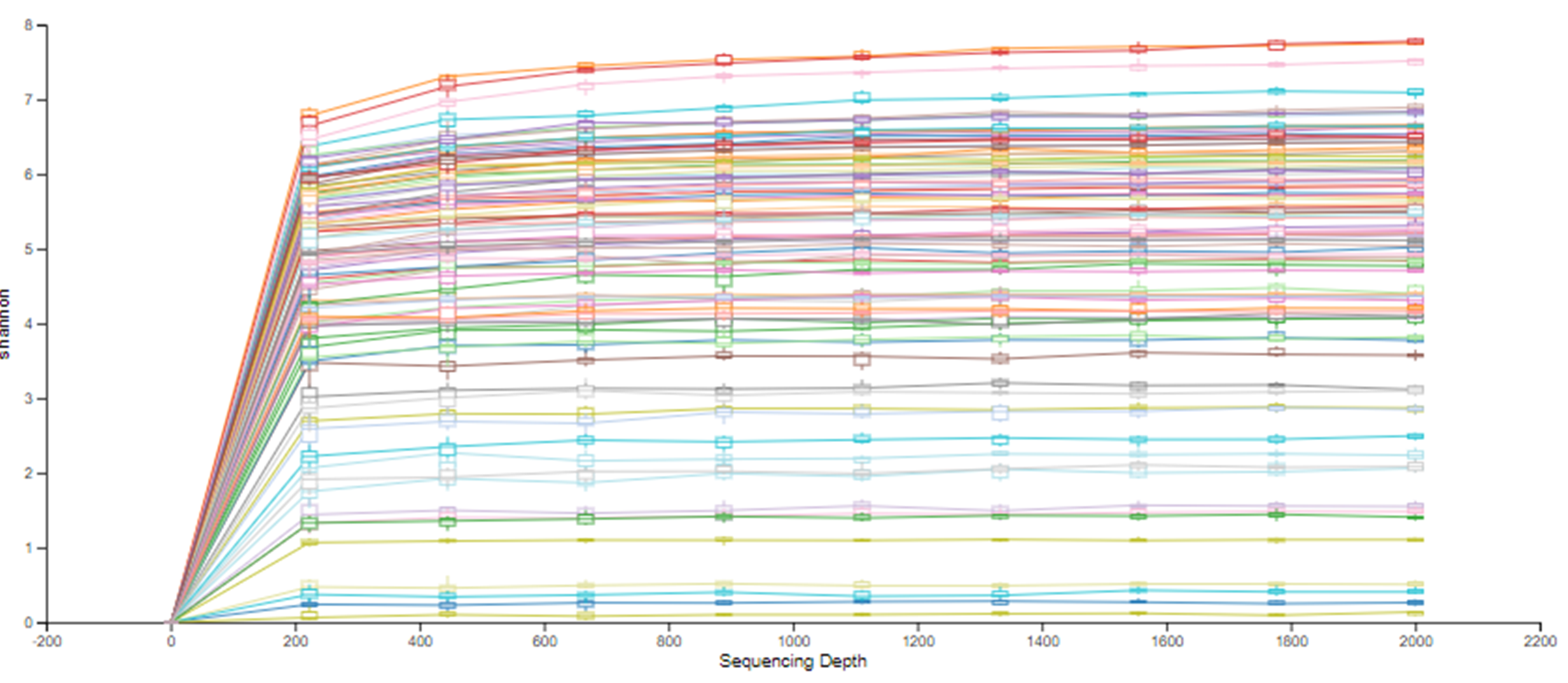


**Figure S2.** Rarefaction curves were used to estimate richness in the Shannon index Figure 16. S rRNA. The vertical axis shows the Shannon index observed and the number of sequences per sample is shown on the horizontal axis.
