# Supplementary figures and images for: What Can the Bacterial Community of Atta sexdens (Linnaeus, 1758) Tell Us about the Habitats in Which This Ant Species Evolves?
Source: Insects. 2020 May 28;11(6):332. doi: 10.3390/insects11060332 (PMC7349130; doi:10.3390/insects11060332)

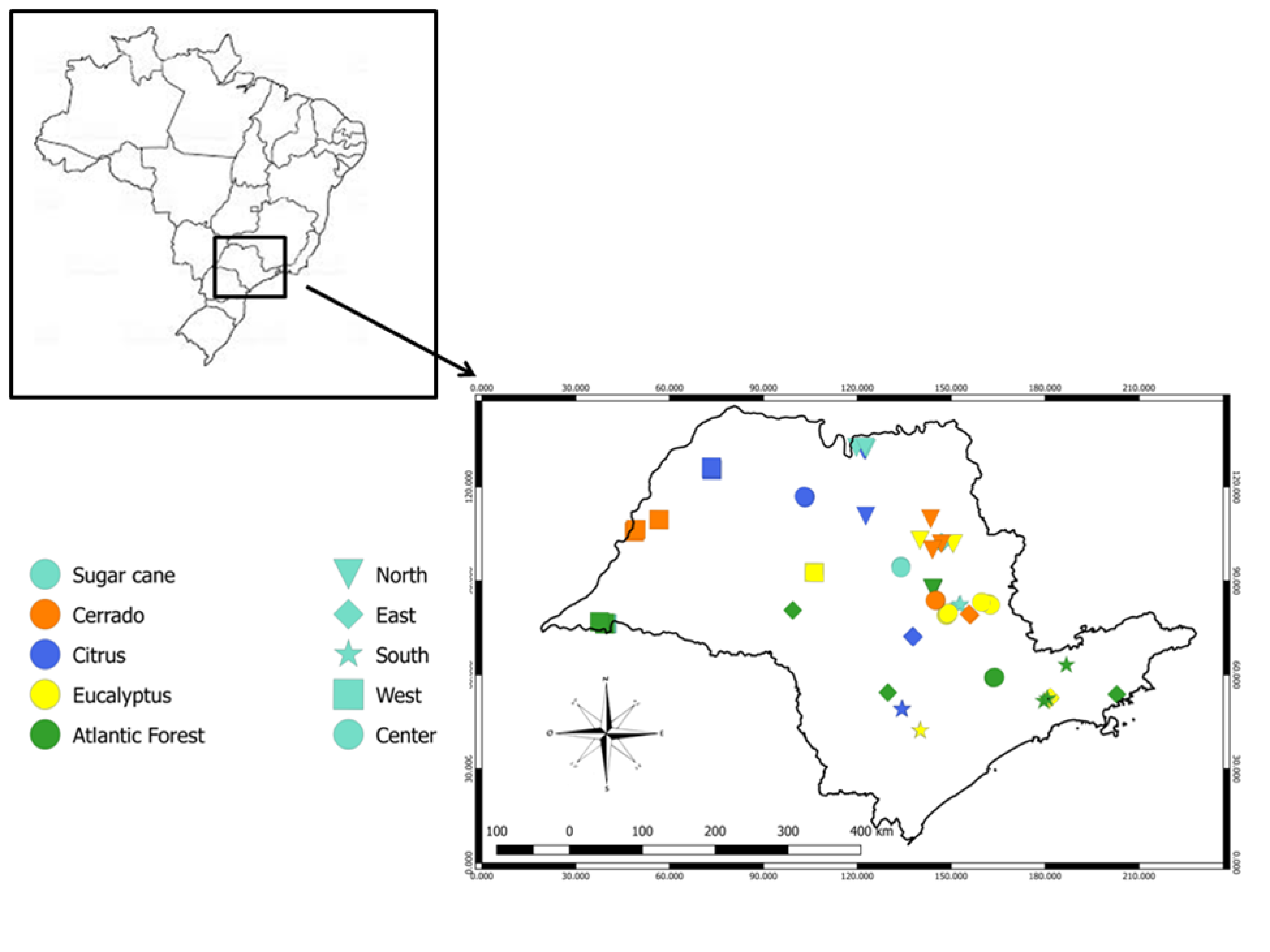


**Figure 1.** Location of*Atta sexdens* collection areas in São Paulo State, Brazil.

Supplement: Supplementary file 1 [file insects-11-00332-s001.zip › insects-751960-Supplmentary Materials/Figure S1.docx]

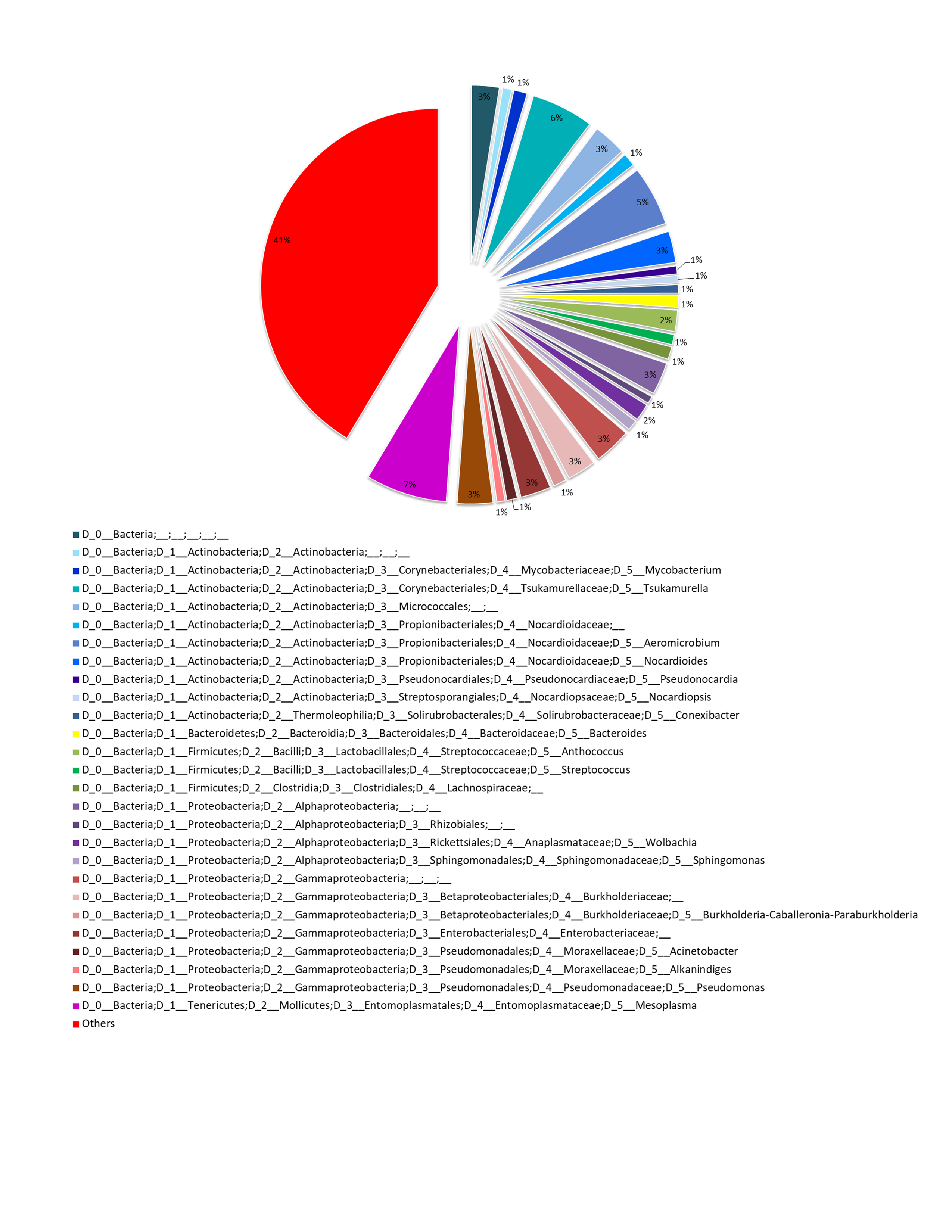


**Figure 3.** The overview of bacteria relative abundance recovered from this study of *Atta sexdens.*

Supplement: Supplementary file 1 [file insects-11-00332-s001.zip › insects-751960-Supplmentary Materials/Figure S3.docx]

**
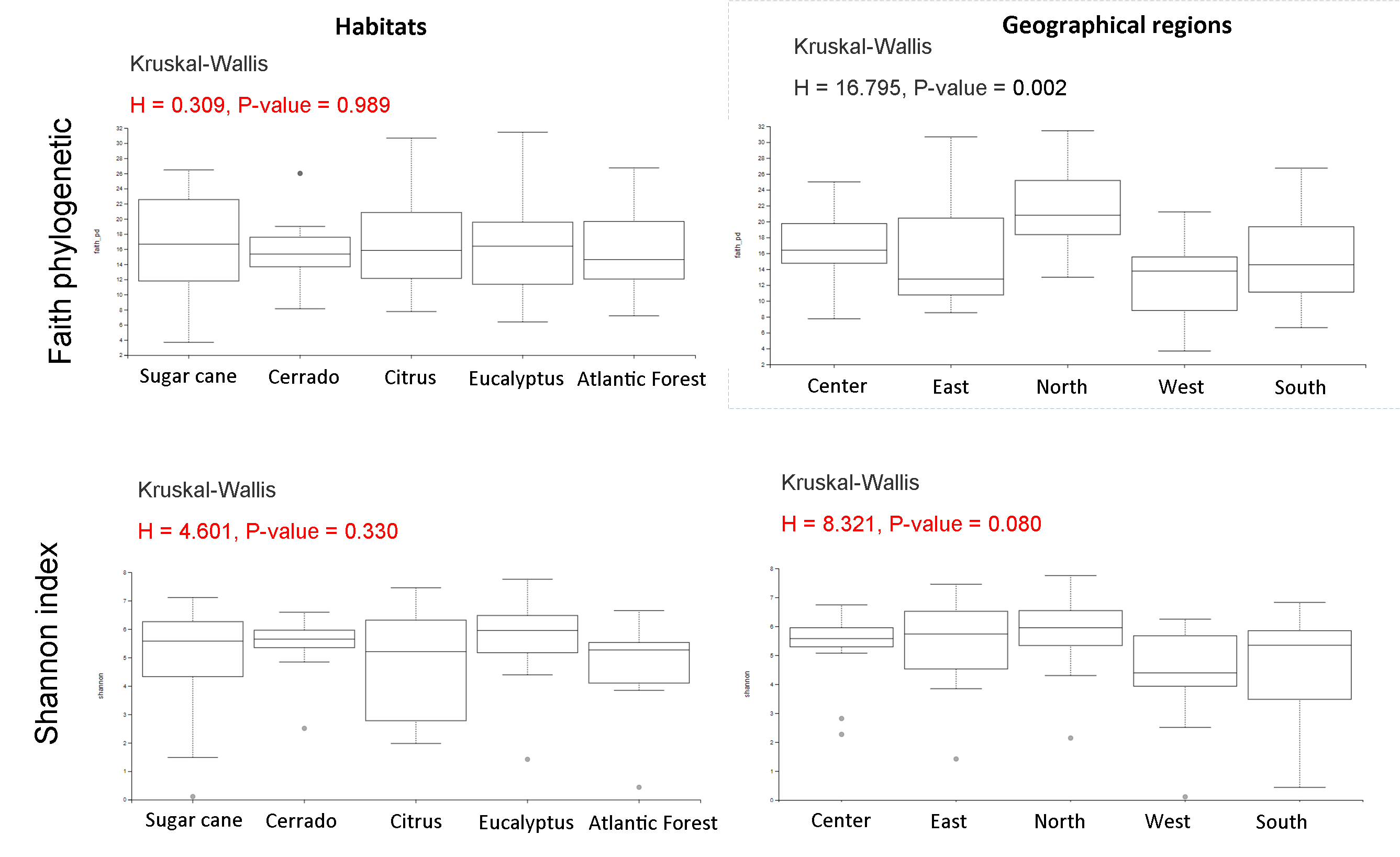
**

**Figure S4.** Alpha diversity associated with *Atta sexdens.*

Supplement: Supplementary file 1 [file insects-11-00332-s001.zip › insects-751960-Supplmentary Materials/Figure S4.docx]
